# As Facts and Chats Go Online, What Is Important for Adolescents with Type 1 Diabetes?

**DOI:** 10.1371/journal.pone.0067659

**Published:** 2013-06-21

**Authors:** Sam Nordfeldt, Teresia Ängarne-Lindberg, Maria Nordwall, Joakim Ekberg, Carina Berterö

**Affiliations:** 1 Division of Child and Adolescent Psychiatry, Department of Clinical and Experimental Medicine, Linköping University, Linköping, Sweden; 2 Center for Medical Technology Assessment, Department of Medicine and Health Sciences, Linköping University, Linköping, Sweden; 3 Division of Paediatrics, Department of Clinical and Experimental Medicine, Linköping University, Linköping, Sweden; 4 Paediatric Clinic, Vrinnevi Hospital, Norrköping, Sweden; 5 Public Health Unit, School of Life Sciences, University of Skövde, Skövde, Sweden; 6 Division of Nursing Sciences, Department of Medical and Health Sciences, Linköping University, Linköping, Sweden; Edinburgh University, United Kingdom

## Abstract

**Background:**

Continued refinement of resources for patient information, education and support is needed. Considering the rapid development of new communication practices, the perspectives of young people themselves warrant more attention using a wide research focus. The purpose of this study was to understand information-seeking behaviours, Internet use and social networking online in adolescents with type 1 diabetes (T1DM). This applied to their everyday life, including the context of diabetes and their experiences and need of contact with T1DM peers.

**Methodology/Principal Findings:**

Twenty-four adolescents aged 10–17 years with T1DM were recruited from a county hospital in the south-east of Sweden. Qualitative data were obtained using eight focus groups, wherein each participant engaged in a 60–90 minute video/audio-recorded session. The focus group data were transcribed and analysed using qualitative content analysis. Some demographic and medical information was also collected. The three main categories that were identified; Aspects of Security, Updating, and Plainness and their sub-categories gave significant information about how to enhance information retrieval and peer contacts related to T1DM. Regarding the persons' information-seeking behaviour, Internet use, and use of social media some differences could be identified depending on gender and age.

**Conclusions/Significance:**

Sensitivity and adaptation to users' needs and expectations seem crucial in the development of future online resources for adolescents with T1DM. To start with, this could mean applying a wider range of already existing information and communication technologies. Health practitioners need to focus on the areas of security of information and communication, frequency of updating, and simplicity of design-*less is more*.

## Introduction

Modern type 1 diabetes (T1DM) treatment is based on patient education, active self-care and multiple-dose insulin using new technologies [1]–[3]. This has facilitated a high quality of life and long-term prognosis has improved, but a large proportion of adolescents are still at risk of acute and long-term complications [4]–[7]. The management of their condition requires daily intensive self-care, including numerous decisions about adaption of treatment to their present habits and activities in life [8]. Modern care supports adolescents with T1DM in gradually becoming their own treatment experts, increasing their own responsibility over time [1], [2], [8]. However, from their perspective, improvements in care are needed regarding patient information and access to services [9]–[11].

For adolescents, the Internet has become an important, trustworthy and valued tool to get information on various health-related issues that otherwise might be difficult to obtain [12]–[15]. In a recent survey from Sweden, from the age of 12 years a majority were already using social networking features online, and at the ages 12–15 years, 91 percent had searched for answers to questions on health issues [16]. However, few paediatric studies provide evaluation of implemented interventions using health information technology [17]. Internet-based interventions may improve access to health services, patient education and quality of care, and have been reported to influence adult diabetic patients' health care utilization, behaviour, attitudes, knowledge, skills, and, to some extent, metabolic control [18], [19]. Positive attitudes towards communicating on the Internet have been reported from adolescents as well as young adults with T1DM, and their health practitioners [10], [20]–[22]. Considering the rapidly evolving new communication practices, a wide and technology-independent research focus is needed. Aspects and voices of young people themselves warrant more attention in the development of future resources [11], [20], [22], [23].

### Aim

This study of adolescents with T1DM aimed to understand their information-seeking behaviour, Internet use and social networking online. This applied to their everyday life including the context of diabetes and experiences and need of contact with T1DM peers.

## Methods

### Ethics statement

This study was performed in accordance with The Declaration of Helsinki and The Swedish Act concerning the Ethical Review of Research Involving Humans (SFS 2003:460). After receiving an information letter from researchers, verbal consent was given by next of kin to a nurse, and recorded in the clinic. Subjects and next of kin were informed in the letter about confidentiality and the right to withdraw without explanation. This was repeated by the researchers through personalized conversations with eligible participants, thus confirming their consent prior to them entering the interview room. The researchers who collected data (TÄL) and performed the analysis (TÄL, CB) had no other relation to the participants and their care process. Furthermore, all data were sampled and analysed anonymously; no consent forms were used for participants or next of kin. Each participant was compensated with a cinema ticket. This protocol was approved by the Regional Ethical Review Board of Linköping, Permit Number: 2011/167-31.

### Sample and setting

Due to the Swedish healthcare system, the study base was a complete geographic population of adolescents with type 1 diabetes (T1DM) living in the catchment area of a county hospital in the South-East of Sweden. A consecutive sample of adolescents aged 10–17 years with T1DM visiting the hospital for a scheduled medical check-up were eligible.

A strategic sampling was made based on their age and gender. The sample included a total of 24 participants; 11 girls and 13 boys. They were allocated into eight groups according to their gender and age: 10–11; 12–13; 14–15 and 16–17 years. The adolescents reported taking 2–7 insulin doses and 0–6 blood glucose checks daily, recent HbA1c 38–91 mmol/mol (DCCT 5.6–10.5%) and diabetes duration 0.5–11 years. All participants were in compulsory or secondary school, depending on age, and everyone had Internet access at home.

Considering the exploratory aim in a rather new field the study had a qualitative inductive and descriptive design. Focus group discussions allowing researchers to examine the participants' point of view as they shared their experiences were used [24]. Obtaining data from eight focus group discussions provided rich and broad information that could help the researcher clarify and understand complex phenomena [25]. Focus group discussions are one form of research where researchers can learn and understand a great deal without knowing exactly what questions to ask.

### Procedure

The adolescents were invited by a posted information letter to participate in the focus group interviews in connection with a scheduled regular check-up with the diabetes team. The interviews were held in a physically familiar hospital context but with no clinical staff present.

An interview guide was used to ensure that the same basic lines of inquiry were pursued with each group interviewed. Questions focusing on information-seeking behaviour, Internet use and use of social media in general, and related to special aspects of diabetes were asked during the discussion. Questions also focused on the participants' experiences and need for contact with other adolescents with T1DM (Table 1). Two current Swedish websites targeting young people with diabetes were demonstrated at the end of the discussion [26], [27]. Each session lasted between 60 and 90 minutes and was moderated by the second author.

**Table 1:** Examples of focus group guiding questions from the interview guide.

When you need to know something, what do you do to get the answer?
Are you a member of any online network? If so, could you tell me about the activities?
Have you ever asked a question on any site on the Internet? If so, what is important when asking?
What is your experience of open websites in contrast to websites with login etc?
What is your experience of contact with other persons who have diabetes?
After demonstration of two websites for youth with diabetes, could you tell me:
-what you experienced as good/important/less important etc?
-what you consider should be changed in order to make the website interesting?

Trained study personnel listened and transcribed each video-audio-recorded focus group session. Information about body posturing, tone of language, facial expressions and interactions were also noted.

### Data Analysis

Considering the exploratory aim of the study, qualitative content analysis was used to code and analyse the transcribed text from the focus group discussions, [28], [29] since content analysis can be applied to transcribed interviews, texts, narratives, letters, documents, protocols and media [30].

Initially the analysis was performed by two of the authors (CB, TÄL) independently. First, all material was read by each of them so they could achieve familiarity with the contents and identify answers given to the focus group session questions. Thereafter the material was read and statements with similarities were clustered and summarized into tentative categories. The tentative categories with all respective statements were reviewed in detail. Unclear statements were explored with respect to the original context.

In a second phase, for reliability, CB and TÄL performed open comparisons. Before the sessions they both read all the primary data and the material emerging in the analysis. Any discrepancies were resolved through discussion. Through iterative in-depth discussions with stepwise re-categorizations and repeated validations vs. the complete primary data, a more logical and complete structure gradually emerged. Thus all sub-themes and themes were validated through repeated systematic reviews of the material.

In a later and final stage of validation, the complete sentences from the original text materials were again reviewed in their original context and condensed into final categories, including some final adjustments. Thus all the categories were validated through systematic reviews of the material.

Both apparent and latent content were considered important. To confirm and illustrate the categories and subcategories selected, quotations related to the respective categories were presented through the work process. The quotes used in the results section were selected to illustrate themes emerging from all focus group statements.

The risk of bias due to the authors' preconceptions or potential expectations was prevented as far as possible through the repeated validations vs. the primary data and the in-depth supervision sessions in the author group.

### Data storage and publishing

In accordance with the Linköping University policies, the data are stored without public access. Manuscript preparation guidelines were obtained from PlosONE and COREQ. The results have not been published elsewhere.

## Results

Regarding the adolescentś information-seeking behaviour, Internet use, and use of social media some differences could be identified depending on gender and age. For children aged 10 or 11, information-seeking behaviour was to turn to parents. As children grew older the Internet was an important tool for getting information. However, in the context of their T1DM, teenagers 16 to 17 years of age still claimed that parents or other significant adults were their main source of information.

The Internet use was not a big issue when the child was aged 10 or 11. Time spent on the Internet was about 30 minutes a day. Mostly, the activity was playing games and the interest areas were determined by gender. Even the age group of children aged 12 or 13 years varied by gender and interest when using the Internet.

Time spent on the Internet was in general increasing due to age, but there was a wide variation between individuals. The boys and girls aged 14 or 15 used the Internet every day for several hours for communication and for getting information. The 16 to 17-year-old boys spent not more than one hour a day using the Internet, due to other interests such as various sports but also homework. The girls in this age group mentioned a more urgent need to chat, comment or look at what others were doing on the Internet, at least for some hours every day.

All participants in the groups mentioned had in some way communicated via social media; getting information, making contact with people, sharing experiences or just making new friends. A majority of the participants had never visited the demonstrated websites before.

The need for contact with other adolescents with T1DM varied immensely. Some wanted contact with persons like them and others wanted to be someone special. Some thought that the contact with other adolescents was the interesting issue, not having diabetes; they just wanted to be like anyone else. The most important thing about having a friend was the person; not that the person had T1DM. But it was valued that their friends knew about their diabetes if something happened.

Three main categories were identified/constructed during the analysis and labelled as aspects of security, updating, and plainness. Each main category included a number of sub-categories at varying levels of abstraction, and the categories were linked together by the underlying meanings [28], [29]. Quotations shown to illustrate and confirm the categories include the moderators' questions in italics.

### Aspects of Security

The first main category concerns different aspects of security, a subject that was often mentioned by the participants. Security means that they as Internet users can be in a state of being secure. The analysis identified the three sub-categories of seriousness, integrity and identity.

The adolescents mentioned the importance of the impression they received when entering a website. In the context of T1DM it was especially important that they had a serious and trustworthy impression.

-*Is this a site which you think it seems that you can trust? (Researcher)*

-Yes (L)

-Mm (M)

-*How do you notice this then? (Researcher)*

-We've got diabetes, so we know… (L)

-It is a good design… (M)

-Yes (L)

-Those that are false usually just… (M)

-Mm (L)

-*So you get an impression through the design? (Researcher)*

-Yes (L)

-*Mm, is there anything else on the site, making it feel reliable? (Researcher)*

-It contains a lot of facts (M)

-Yes for example “What is diabetes”, then you get… (L)

-And there are points below… [a sub-menu] (M)

-Yes, so that you, for example can show your classmates if they don't understand (M)

(G3, Girls 12–13 years)

Seriousness concerned the trustworthiness and reliability in the information given on the website. They said it was important to know who was behind the information and if the information could be considered as given by professionals or persons with proper knowledge. In the context of how to assess the seriousness of the website some said:

-Well…if you feel certain about what kind of website it is, who's behind it, then you can trust that there's no false things there, then that's good, and maybe you don't have to call the hospital and ask questions (M)

-Yes (L)

-*So you could get answers? And are there any drawbacks with such a site? (Researcher)*

-No, I don't think so (M)

-Noo… (L)

-*No… What makes you not enter such a site? (Researcher)*

-Sometimes because you don't have time (L)

-Or you don't have anything to do there (M) //

-…*do you have ideas? (Researcher)*

-Mm, make it interesting both to us young and maybe slightly older ones (M)

-Yes and maybe inform schools (L)

(G3, Girls 12–13 years)

Many expressed a wish to behave with caution online, to only share opinions and thoughts with those who had their approval, mainly persons they had met and knew in person. Integrity meant an unimpaired condition grounded in honesty. It could be seen as an emotional aspect of security dealing with thoughts about risks and potential situations. Discussions were on general subjects but concerned, to a large extent, the risk of getting in contact with persons who present themselves as being other than they actually are. The subject mainly came up among the girls.

-*Can you see any advantages or disadvantages with meeting on the Internet or in person? (Researcher)*

-You easily misunderstand each other on the Internet, like when you write something… (I)

-Like, if you have made an appointment through the Internet to meet somebody, it may not be the person you expected (B)

-*Like if you make an appointment with somebody that you should not have met? (Researcher)*

-Yes (B)

(G2, Girls 10–11 years)

-It could be a psycho, it could be any person, you never know, it's quite uncertain (A)

-One must think about who to add (E)

-Mm (S)

-Exactly, maybe it's different if you know someone who might know this person through someone else (A)

-Mm (E, S)

-Maybe a little different to add, but no, …no one I don't know, I might, it could be a paedophile, you never know really. That's what's so scary (A).

-Exactly (E)

-Yeah (S)

-He might call himself something… (E)

-Yes exactly, or take a photo of someone else, and then swap it with is own so you

think that it is… (A)

(G1, Girls 14–15 years)

However, opinions about anonymity was most clearly expressed by some of the fifteen-year-old boys, and showed that anonymity was regarded as cowardice. Even so, in several groups the opinion was that anonymity could be a good thing when asking certain questions.

-*Well,…and what do you think about websites being closed or open…do you know what I mean?… when you're entering Facebook for example, then others can see that you are online (Researcher)*

-It's not completely so, I could be sitting at my computer, but somebody else could enter Facebook without knowing if it's me (J)

-*Yes, that's true. But what do you think, is there any point to open or closed, to a website being open for everybody or only those logging in? (Researcher)*

-I think you ought to log in (J)

-That's how it is, the worst there is if there are anonymous persons there… if you're serious yourself about something and an anonymous person comments one gets angry, the other one could write just about anything because the anonymity shelters him (F)

-On Facebook you have to register with your name and so on (J)

-*Mm, so you think that's being a coward? (Researcher)*

-Yes (F)

-Mm (J, Ph)

(G1, Boys 14–15 years)

-*What do you think about things you write on the Internet, should it be visible to others or non-visible? (Researcher)*

-Well, yes I think so, well though a blog, I have a blog… you can write anything there (B)

-*But is it okay for anybody to read it? (Researcher)*

-Yes (B)

-*Is there anything you write on the Internet that doesn't feel okay that everybody reads? (Researcher)*

-No, but you see most websites, if you're creating a website which you don't want everybody to see…only just friends, then you can arrange it with passwords. But I don't think so (B)

-*What do the rest of you think, should it be open or closed or…? (Researcher)*

-Well it is different… (S)

-*It's different? (Researcher)*

-It depends on what it is (S)

(G2, Girls 10–11 years)

Identity is about personality or the state of being. Identity touches upon integrity, but they differ. Integrity could be seen as a function that contributes to protecting identity, and that makes anonymity possible. Most participants preferred open access to the website, with no login, since it seemed relatively unlikely that non-serious persons would visit websites concerning diabetes. Another aspect was the thought that it would be too much trouble having a login.

-The website here looks serious, if it should be some strange person, or for instance a paedophile, I don't think that he would enter a diabetes website and look there. I rather think that those who enters a diabetes website are those interested in diabetes as a subject (A)

-If you're not a diabetic, you might not even know about the website (E)

-No, exactly (A)

-The nurse showed us this site (E)

-Yeah (S)

-Mm, I think so too (nodding) (A)

(G1, Girls 14–15 years)

However, most participants advocated open access to the website, combined with a login to parts of the services offered on the website, meaning interpersonal services such as chat.

-*If you have a website where it's possible to ask questions and chat, should it be open for others to see, or should it be closed, or both, so you can choose if you want others to see or not? (Researcher)*

-Mm, in that case choose as on Facebook (M)

-Yes (L)

-Mm (M, L)

(G3, Girls 12–13 years)

### Updating

Updating is about what could be seen as the continued existence of a website in the sense of keeping the visitors interest through continuous adjustment of the website. It is also about addressing a message about presence and interest behind the website, to demonstrate that this is a living website. This main category, updating, consists of three subcategories: news value, facts, and eye-catching. The two first sub-categories are more closely related to each other, but not intertwined.

-Like there ought to be news. Well there is research there (F)

-*But we have found the updated research? (Researcher)*

-If you had some kind of updating, showing this is new (Ph)

-I can imagine that this information has been here since the site was new right? (F)

-*I don't know, no… (Researcher)*

-I guess so… cause mm… it is, then make it a blog instead, research, it's like a blog, date, topic, like that (F)

-*It would be better with a blog? (Researcher)*

-Yes like a blog somehow… (F)

-*Is it like when you get the feeling if it isn't new it won't be as interesting to read? (Researcher)*

-Yes (F)

-*Mm (Researcher)*

-Yes (J, Ph)

-*What then if you enter several times and it remains the same? (Researcher)*

-Yes in the end just no, there is no news here, so you don't care about it anymore… (F)

(G1, Boys 14–15 years)

News value is concerned with current event or a recent happening. In other words, news value is about getting information not known before. The participants expressed a wish to get information about news in the area of diabetes; news with an emphasis on means of assistance, but also reports about inspiring achievements by persons with diabetes, e.g. a young person having T1DM who has bicycled through Europe.

-…that's old news like, nothing you care about… (F)

-*No, okay (laugh) (Researcher)*

-KP [a magazine for children] alike (J)

-It's type of old news somehow (F)

-Yes (J)

-*So, it's important to… (Researcher)*

-Nothing you care about (F, J agrees)//

-But that's quite cool… (F)

-*What's cool about Daniel Larsson cycling through Europe? (Researcher)*

-That's cool (F)

-Yes, that he managed to make it (J)

(G1, Boys 14–15 years)

-*It might be a difficult question, but what's the ultimate site to you?*

-I think it looks fine like this, it should not only be about one thing, there should be different tips and links and… (A)

-Yes (E, S)

-Yes, but all this (A)

-And some food tips (S)

-*Did you say food tips? (Researcher)*

-Yes (S)

*Are those here? (Researcher)*

-Plenty (A)

-*Yes (Researcher)*

-And there is news, what happens in the body, sexuality, and lots of different things (A)

-It is… (E)

-…about insulin and medical devices, and about food and how you're affected by things… what you ought to think about, not to smoke, and with diabetes, don't drink and such stuff (A)

(G1, Girls 14–15 years)

Facts are about verifiable information, a thing known to have happened, something found in reality, for example the risk of complications. The participants expressed a desire to get information about existing and new research in the area of diabetes, but also to find out about future research and possibilities and visions of the future.

-*…do you find it interesting to get information about long-term complications? (Researcher)*

-Ehm… so that you get to know something about what's going to happen (M)

-Yes, about what's going to happen when I get a little older, like you 80 years (laughs, turns to M) (L)

-*Mm…* (laugh) (*Researcher*) //

-*You think it* [a website, ref 26] *seems good? (Researcher)*

-Yes. And then on long-term complications that comes up [pointing at a facts menu] so you won't have to go through it all, so you get facts at once (M)

-Yes (L)

(G3, Girls 12–13 years)

-Well like news about research and questions about diabetes (Ph)

-*Mm (Researcher)*

-It would be fun to know just, yes, that there will be the remedy for diabetes within 15 years and…it is… they have been talking a lot about finding a certain remedy to regain your insulin production. It disappeared for me one year after being diagnosed with diabetes (Ph)

-*Mm (Researcher)*

-Like that (F)

-Nasty thing for you (J)

-Yes, …imagine for Ph to be like cured but I can't cause I've had it too long

-Like that (F)

-*Well, so you're interested in research or specific questions then? (Researcher)*

-Yes (F, J)

(G1, Boys 14–15 years)

Eye-catching is about something being striking in appearance. Related to design and layout, the emphasis here is about what catches the visitors' eye and attention, and gives the impression that something new has happened since the last visit to the website.

-And here are active threads (F)

-*Yes (Researcher)*

-And you take that list and put it on the site (F)

-*On the site there (Researcher)*

-And if somebody has started a new thread or commented on somebody else's you can see that here, then that's an active thread… (F)

-*So it feels like something new, is that the point? (Researcher)*

-Yes like then it’s something popular, a hot discussion or something… (F)

(G1, Boys 14–15 years)

### Plainness

The third main category is about plainness, meaning that something is straightforward, understandable, almost obvious. It could be plainness in the sense of intelligible layout and content of the website. The category consequently consists of three sub-categories: layout, content and congeniality.

Layout is about the manner in which anything is laid out e.g. arrangement of text and pictures. In the discussions, layout was connected to both content and congeniality in that it helps the content to be understandable and easier to apprehend. A layout considered to be congenial can encourage site visitors to read the content; through awakening their interest. However, the focuses here were mainly on function, while congeniality was to be seen as an expression of the power of attraction.

When two websites for diabetic youth [26], [27] were demonstrated almost every participant said that the layout of one site [26] was good. Lucid, well arranged, and easy to grasp are illuminative examples of what were considered characteristics of a good layout.

-It's a pretty good design (F)

-Yes the design looks good (J)

-Well, the design does a lot… (Ph)//

-*Yes it does (Researcher)*

-Else it's… it doesn't feel the same as that other site (Ph)

-*Young diabetes? (Researcher)*

-Yes that one seemed a little more ehh… (laugh) …OK but difficult to find things (Ph)

-That was the ugliest site I've ever seen (F)

-Mm (J)

(G1, Boys 14–15 years)

Content is about volume and substance, but also accessibility, meaning comprehensibility of the information. The content of one site [26] was described by the participants as extensive. Besides suggestions about games on the website, mostly among boys, no need for additional services was expressed.

Some contents were given particular attention. Boys, in particular, thought that the heading “Research” was interesting, containing research about diabetes. Other headings described as interesting were “Blogs” and “Open forum”. A somewhat greater interest in blogs was noticed among the girls and in the forum among the boys.

The younger girls spoke about the importance of content written in an understandable way. They also suggested that texts should be adjusted according to age or capacity to understand.

-But like this, there shouldn't be too much text, because then I think people will

get tired (M)

-*So you think there shouldn't be too much text on a website? (Researcher)*

-No, short and good (M)

*Does this count for all written information… should answers to questions be short too? (Researcher)*

-Ehh (M)

-That depends on the question (L)

-Yes (nodding) (M)

(G3, girls 12–13 years)

-I won't go in, or if I enter a site looking like that, I leave (giggle) (B)

-*Aha, so it's the look of the website that makes you interested? (Researcher)*

-Yes (B)

-*What do you others think, what makes you interested? (Researcher)*

-I want like…I want it to be colourful and funny… (E)

-*Do you others agree, or do you have other thoughts? (Researcher)*

-Well, maybe a little more children's facts so to speak, with words that are easy to understand (I)

-What's the name, Young diabetes, well then the other one is for younger diabetics (giggle) (E)

-It should contain… (E)

-Some facts (B)

-*Facts… (Researcher)*

-Yes plenty of tips and facts could be there, maybe quite a few tips on which sports to try if one can't move so much (E)

-*Yes, good sports? (Researcher)*

-Yes that are good for diabetics, because otherwise you may start with a sport where you don't move much (E)

-*Mm… mm… yes… something else… (Researcher)*

-Like others, like Diabit [26] like blogs so you can get tips and so on… (B)

(G2, girls 10–11 years)

Congeniality or power of attraction is about experiencing suitable or agreeable websites. The participants expressed the importance of a rich colouring of the website. They also specially emphasized the need for a moderate quantity of text. Colours and a moderate quantity of text were seen by some of the participants as signs of youth, while greyness and a large quantity of text was seen as a signal of adultness.

-I don't know if I would have entered here, it looks so boring (M)

-*Noo (Researcher)*

-It doesn't look very fun (M)

-It looks so bo-oring (L)

-*It looks boring. Is it the look of the website that looks boring? (Researcher)*

-Yes (M)

-Yes (L)

-*So here you wouldn't look any further? (Researcher)*

-I would rather suggest it to an adult (L)

-*You think it's directed to adults? (Researcher)*

-Mmm (M)

(L nodding)

-That one looks much better (M)

-Yes (L)

-*Could you tell what it is that makes this one look better? (Researcher)*

-It's more colourful… (L)

-*Coloured, yes (Researcher)*

-And more organized (M)

-Yes (L)

-*Okay (Researcher)*

-So that you find things easier (giggle) (M)

(G3, Girls 12–13 years)

In summary the three main categories; aspects of security, updating and plainness and their sub-categories gave some significant information from the perspectives of adolescents with T1DM using the Internet.

## Discussion

Even if many adolescents with T1DM are frequent Internet users, the challenge remains for health practitioners to develop and iteratively refine systems which are attractive and useful to young people [17], [19], [20], [22], [31], [32]. The phenomenon of attrition-losing many of the users after a certain period of time-applies to a varying extent to most e-health interventions [33]. Adolescents as well as adults with diabetes may visit various online forums for social support, information, advice, and to share experiences [11], [15], [20], [22], [34]–[36].

Security comprises feelings of integrity in opinions and thoughts shared only with approved persons, and it is received through functions protecting the Internet user's identity. However, in the context of the adolescents' T1DM, an important part of the security was the trustworthiness and reliability of the information given on the website. This form of security was provided through having knowledge about the professionals behind the site. The same applies to adults; perceiving a site as secure appears fundamental [37].

The most common response, regardless of age, to the question about where they sought information, proved to be parents or other significant adults, including the clinical staff, in the context of T1DM. A majority also claimed to know and to have met in person most of their Internet contacts, and they regarded it as important to have confidence in and to be linked to one another.

Thus the results point at the importance of confidence in relationships for maintaining seriousness, integrity and identity. It might be that online information, especially in the context of a serious illness such as T1DM, needs to be paired with contact with known and trusted persons.

Interaction on the Internet, in the context of their illness and elsewhere, is not limited to such known persons; e.g. some adolescents may visit a discussion forum rather than contacting their clinic [34], [38]. Thus, the issue of security equally applies to social networking for diabetes-related issues and to relational and interpersonal contacts with non-diabetic peers [37].

The adolescents in this study confirm that it is important for site editors and practitioners in charge of a health information site to be active in updating the site and otherwise keeping it alive. As found in previous studies on young people with diabetes, some hold high expectations of a living online community [20], [22]. Usually few users contribute actively, whereas a majority of visitors are passive, not commenting themselves [35].

The latest news from the diabetes research field, and other news, for example about technical devices, were other areas described as important to keep updated. The former applied especially to boys aged 14–15, and the latter to girls aged 14–15. This is consistent with previous case studies where the respondents reported about experiences concerning useful facts, updates and current research in the field [20], [22].

Our participants spoke about the importance of the information/content on the website being accessible to them. The younger girls (aged 10–11) emphasized in the context of their T1DM, the need for the language on the website to be adjusted to different age groups or linguistic levels. The older boys (aged 14–15) emphasized the amount of text should be adapted to adolescents and pointed out specific areas of interest such as diabetes-related research.

When information is mediated by dialogues online, the concept of intergradation could be useful to reach a deeper understanding of how to exchange information. Based on observations of nurses and children, intergradation has been described as how the phrases used merge gradually with one another, maintaining the integrity of all, through dialogues that constitute unbroken sequences of information exchange [39], [40]. Thus intergrade is about sensitivity and adaptivity to each person's needs; which requires being clear and plain in the information exchange.

Regarding other factors that may influence the decision to stay on a website, a majority of the respondents spoke of the importance of the site ‘looking good’ and ‘making things easy to find’, the latter being most pronounced among boys 14–15 years. In earlier studies, adolescents and their parents have also emphasized the importance of the appearance of a website, and simplicity in its usage and understanding; thus it is important both to catch the visitor's interest and also to keep it [41].

### Methodological considerations

To enhance participation, we performed the interviews in a hospital setting familiar to the participants. They were informed of the researchers' independence from the clinic before giving their consent to participate, and no clinical staff were present during the interviews. Because qualitative methods were used to gain a deeper understanding of the respondents' perceptions, it is not possible in this study to make generalizations in a quantitative manner.

Altogether, the interviews were a rich source of information even though individual statements were often rather short. It is possible that use of repeated sessions or additional digital communication could have further expanded the body of data.

### Implications

Clinical implementation of Internet-mediated information and support for young people with T1DM need to make use of the rapid developments of the web and the wider world [17], [19], [20], [42], [43], [44]. To start with, this could mean applying a wider range of already existing, widely used, and simple technologies. Contact with the clinical staff when needed is essential. For example, one-to-one advice delivered by a trained peer-advisor and/or diabetes healthcare professional using instant messaging or videophone options may be one such avenue.

The findings of this study need further attention. How can security on a website be shown and recognized by adolescents? Next to parents, the clinical staff proved to be an important and reliable source of information. Thus a reasonable suggestion could be that health practitioners should strengthen the reliability of the website by being openly involved and active in dialogues.

Our participants expected a secure and respectful diabetes-related website. Thus a communication policy defining core values displayed through professionals' attitudes when intergrading online might contribute to trust and security. There are several possible ways to demonstrate updating and enhance security on a diabetes portal for adolescents. On factual content, having the statement “latest updated at …” brings clarity. News and practical hints from local practitioners may be highlighted. Contributing practitioners may show their identities and professional positions. However, the greatest resource for updates and keeping a site alive might even be young users themselves in forum discussions and areas for personal stories and blogs [15], [20], [22], [34], [43].

Finally, it seems to be of a high importance to maintain simplicity, uncomplicated language, and information content adapted to all users' needs. Apart from a clear and plain design structure this also requires an editorial policy including a continuous refinement process for language and information content. Editors and practitioners intergrading in online dialogues with and/or between adolescents might gradually gain a deeper understanding of their perspectives, which would be useful for iterative refinement of information contents.

### Further research

Even if Internet use expands, diversity of experiences and preferences will remain. Thus simple web portal solutions hosting practitioners' factual information, adolescentś and parents' shared experiences, and featuring practitioners who are active in open dialogues seem worthy of further evaluation.

Taking users' perspectives into account throughout the process, including iterative real-world usability testing, seems crucial to ensure that future online health-related services for adolescents and parents meet the users' needs [19], [41], [43], [45]. Our study and others indicate a need for further consideration of age differences as well [46].

### Conclusions

Sensitivity and adaptation to users' needs and expectations seem crucial in the development of future online resources for adolescents with T1DM. Health practitioners need to focus on frequency of updating, security of information and communication, and simplicity of design-*less is more*.
